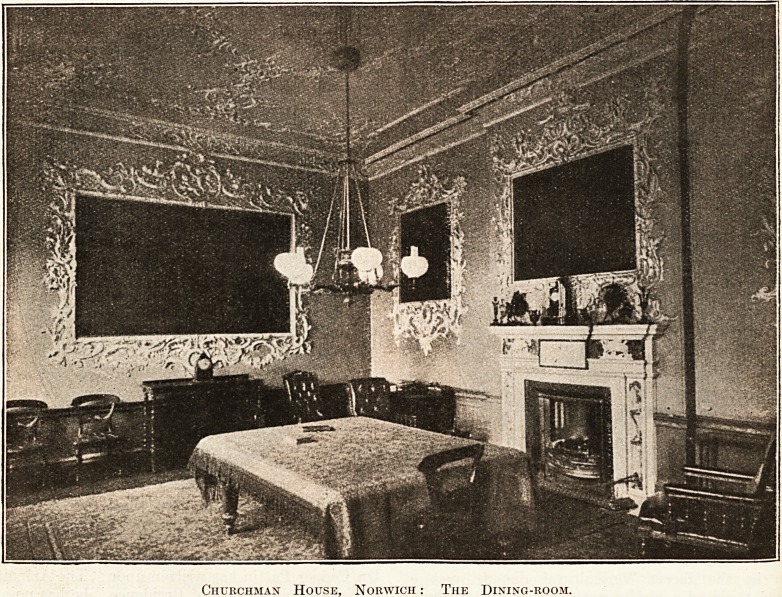# The Public Health: Interviews with Local Authorities—Norwich

**Published:** 1923-07

**Authors:** 


### THE PUBLIC HEALTH.

### INTERVIEWS WITH LOCAL AUTHORITIES.

### X.—NORWICH—THE CITY OF CHURCHES.

(By our Special Commissioner.)

ROBERT KETT, the tanner, in 1549, with a fine instinct for the public health, led a movement of discontent against the enclosure of common lands. He was hanged in chains from the top of Norwich Castle, three hundred of his followers also being hanged in order to point the moral. Nowadays Kett would be far more likely to have found recognition of his public services by being made Lord Mayor of the city. He would, however, have proceeded by more constitutional methods, just as masters and men in the recent agricultural dispute met at Norwich round a conference table at the Bishop's Palace, on the invitation of a wise and kindly prelate, to discuss their differences.

[Coe, Norwich.

Dr. H. Cooper Pattin,
M.O.H. of the City of Norwich.

Mr. Herbert P. Gowen,
Chairman of the Norwich Health Committee.

The city and county of Norwich—for Norwich has the dual status—is fortunate in the Chairman of its Public Health Committee, Councillor H. P. Gowen, who does not allow his business instincts (he is an accountant) to overrule his zeal for the health of the community. He is ready at all times to give a sympathetic ear to the recommendations of the Medical Officer, Dr. H. Cooper Pattin, who knows his Norwich through and through, and clearly loves it well. These two gentlemen received us with much kindness and gave us an interesting account of some of their work and of the health conditions of the city generally.

Health nowadays, Mr. Gowen properly observed, is before all else a housing question. Given some better and more consistent guidance and help from the Minister of Health, on the coming into law of the new Housing Act, it will be found that Norwich will be well prepared with proposals for the betterment of housing conditions in the city, even though a special rate may be involved. The problem here is not created, in the main, as in the manufacturing cities of the North, by the industrialism of the nineteenth century. Norwich is none of your mushroom growths. In an interesting memorandum which Dr. Cooper Pattin has written upon " The Amenities of Norwich as a Place of Residence," he has pointed out that this has been a relatively populous community for over a thousand years, and that records afford evidence that the city has been found to be a salubrious and attractiev place in which to dwell. Evelyn, visiting Norwich in 1671, wrote of " this ancient city, one of the largest and certainly, after London, one of the noblest of England for its venerable cathedral, number of stately churches, and the cleanness of the streets."

Although, therefore, the housing problem is a growth in Norwich having its roots deeper in history than in the case of the modern manufacturing areas, the problem is there ; and it is insistent. A feature of much of the house property in the city is—to use an expression of Dr. Pattin's—its worn-up character, and " we cannot," said Mr. Gowen, " call upon an owner to spend a hundred pounds or more on reparations, however urgent the need, if we know that in a year or so we may be calling upon him to pull the place down." In addition to the problem of the "worn-up" house and that of the general lag of building behind the demand, there is the question of the small dwelling. It was brought to our notice, in connection with the work of the Health Visitors, that 17 per cent. of the dwellings visited possessed only one bedroom, with an average population of between four and five persons.

There is in Norwich one house with which we are immediately concerned which is certainly not worn-up, which, indeed, is in beautiful preservation. It is the early Georgian dwelling in which Sir Peter Eade lived and died. After his death it became the headquarters of the Medical Officer of Health. Churchman House, with its spacious and restful accommodation, is indeed an inspiring office, in which Dr. Pattin may well dream dreams for the health of the community around him. And he will afterwards find a receptive and practical Chairman in Mr. Gowen, whose own staff, in his private business, were seen by us to be occupying offices that seem to be the last word in health equipment. This is not the place for a detailed description of Churchman House, but we have reproduced a picture of the beautiful dining-room. (It is not, of course, now furnished as shown.) The citizen who goes to Churchman House, whether for examination by the Tuberculosis Officer or for some attention in the dental clinic, or on some other disturbing health question, will find the treatment already begun by the restful atmosphere in which he is attended, or in which he is called upon to wait. In the course of our wanderings elsewhere, we have found some dreadful waiting-rooms, uninspiring offices, or premises taken, perhaps, for child-welfare work, which are unlovely in the extreme, however beautiful the work itself or the idea behind it. Other authorities, therefore, please copy the Churchman House idea as far as possible!

Churchman House, Norwich: The Dining-room.

This city of Norwich, of which a fine view is obtained from its famous Mousehold Heath, which is swept by the fresh winds from the East Coast, just a few miles away—stimulating like the city's well-known mustard—is not only the City of Churches, and still jealous, we doubt not, to retain its reputation as the City of Gardens; but it can claim to be a healthy city. Norwich is drier and has a lower rainfall than the country as a whole. The water supply is good. It has a population of about 123,000, with a death-rate of 12·5 per 1,000, and the infantile mortality figure of 72 per 1,000 births compares very favourably with that for most towns. It is also a city of many charitable foundations, ecclesiastical and other, among which pride of place must be given to the work of the Norfolk and Norwich Hospital, with its 220 beds, a centre from which the assistance of trained nurses is obtained, as also from the Home which perpetuates the memory of Nurse Cavell, who was born near Norwich and trained in that city.

In connection with the care of the children, mention must be made of the voluntary labours for years past of Dr. Margaret Boileau at the Infant Welfare Centre. They believe in looking after the babies in Norwich, and a good deal of significance attaches to the simple statement that the addition to the staff of a new health visitor " enables us to have babies completely undressed more frequently and expeditiously." The chairman of the Health Committee clearly finds unwelcome the restraining hand of the Minister of Health where the case of children is concerned ; that is an unsuitable point at which to talk of economy with a capital E, even though, as vice-chairman of the Finance Committee, Mr. Gowen is fully conscious of the need for close watchfulness. Milk, both fresh and dried milk, to nursing and expectant mothers, and for ailing or ill-nourished infants must, in the view of the Health Committee, be supplied generously in a city which might well seek in this way to be second to none, for is not Norfolk a county of rich pasturage, and is it not also the county in which the King has his country home ?

The school medical work of the city, the care of the tuberculous and of those suffering from other forms of disease, laboratory work, and the hundred and one details of modern sanitary administration all receive the constant and unremitting care of Dr. Cooper Pattin and his staff, with the sympathetic and enlightened support of the committee of which Mr. Gowen is the chairman. You may go to Norwich as a visitor to see its stately cathedral, its wealth of churches—including the imposing Roman Catholic Church of St. John endowed by the late Duke of Norfolk—its historic castle, and its old Guildhall and other antiquities. Or, if you happen to live in one of those pleasant clusters of red-roofed villages which help to make, with the streams and meadows and windmills of Norfolk so pleasing a landscape, you may go to Norwich as to your metropolis and your market. In either case you will agree with George Borrow that it " is a fine old city, truly." And if you get something of Borrow's enthusiasm, and add " Who can wonder that the children of that fine old city are proud of her and offer up prayers for her prosperity ? " you may reflect that here, as elsewhere, the ultimate happiness of the citizens, without which prosperity matters little, depends in large measure on their health, and that in the City of Churches prayers might well be offered for those who are working for the health of the citizens.

### Conference on Tuberculosis at Birmingham.

As stated in our last issue, the annual conference of the National Association for the Prevention of Tuberculosis is to take place at Birmingham University on July 12, 13 and 14. The conference will be opened by the Lord Mayor of Birmingham on the 12th, and he will also hold an evening reception in the Art Gallery at 8 p.m. on that day. The subjects for discussion include the Care of Advanced Cases of Tuberculosis, the Work of Tuberculosis Care Committees, and the Relative Prevalence of Tuberculosis among Workers in Different Trades. An evening lecture will be given on the 13th by Professor Sir Robert Philip, M.D., on " The Actual Position of the Tuberculosis Problem To-day " ; a popular lecture on the subject will also be broadcasted.

## Figures and Tables

**Figure f1:**
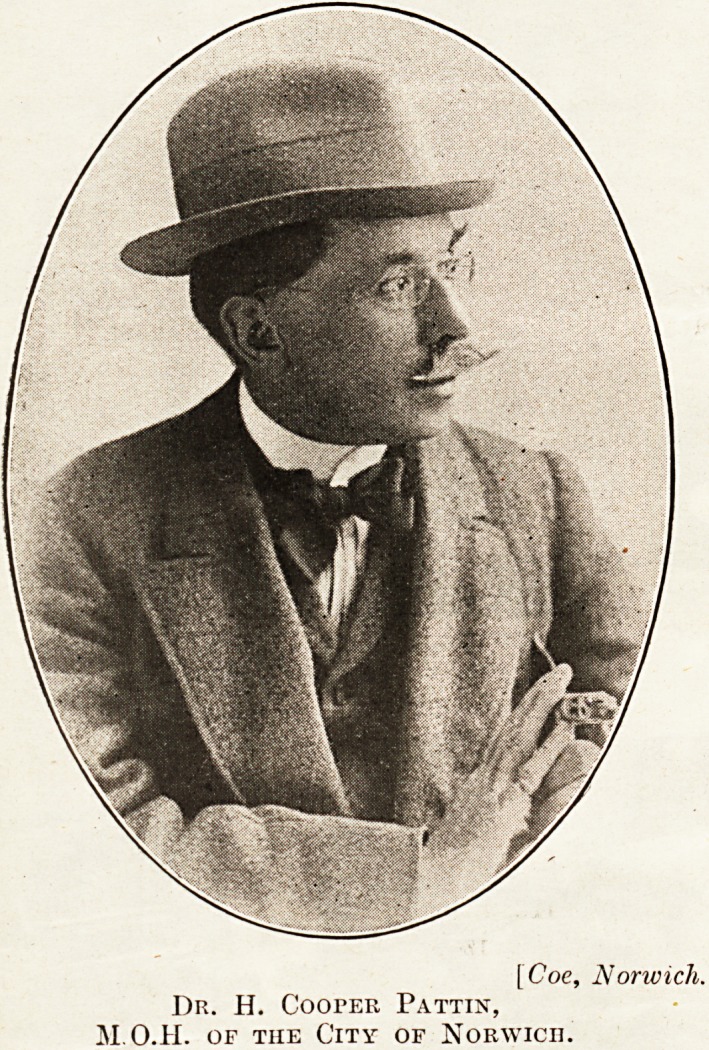


**Figure f2:**
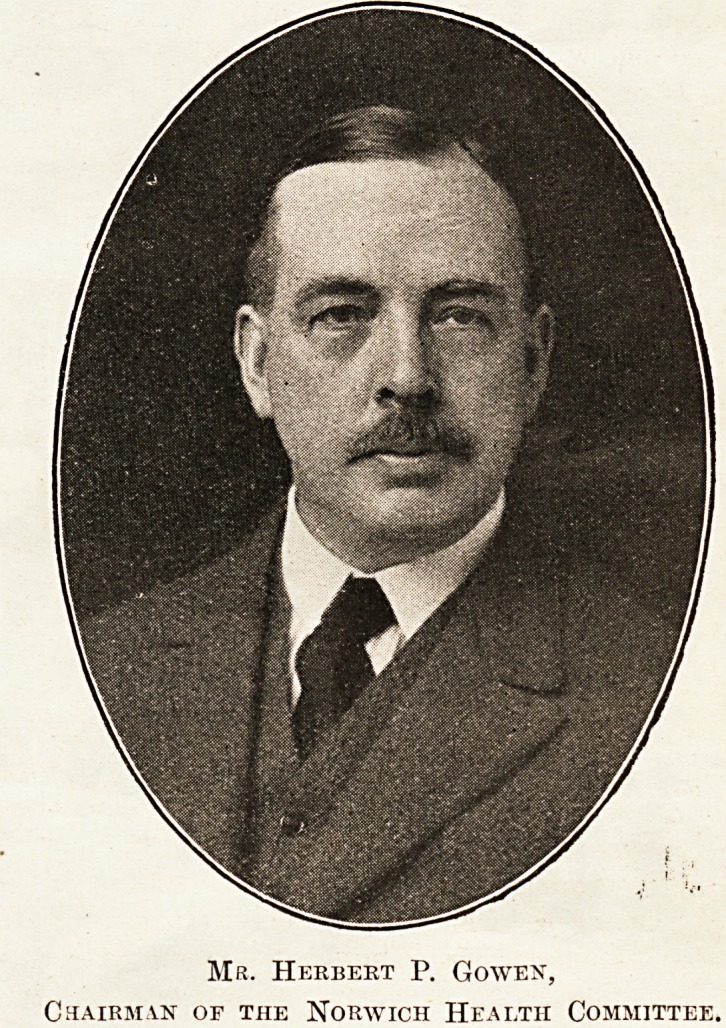


**Figure f3:**